# Fibronectin Regulation of Integrin B1 and SLUG in Circulating Tumor Cells

**DOI:** 10.3390/cells8060618

**Published:** 2019-06-20

**Authors:** Jeannette Huaman, Michelle Naidoo, Xingxing Zang, Olorunseun O. Ogunwobi

**Affiliations:** 1 Department of Biological Sciences, Hunter College of The City University of New York, New York, NY 10065, USA; JHUAMAN@genectr.hunter.cuny.edu (J.H.); michelle.naidoo86@myhunter.cuny.edu (M.N.); 2 Department of Biology, The Graduate Center of The City University of New York, New York, NY 10016, USA; 3 Departments of Microbiology and Immunology, and Medicine (Oncology), Albert Einstein College of Medicine, Bronx, NY 10461, USA; xingxing.zang@einstein.yu.edu; 4 Department of Medicine, Weill Cornell Medicine, Cornell University, New York, NY 10065, USA

**Keywords:** metastasis, circulating tumor cells (CTCs), hepatocellular carcinoma (HCC), castration resistant prostate cancer (CRPC), epithelial-to-mesenchymal transition (EMT), fibronectin, integrin B1, SLUG, major histocompatibility complex class I (MHCI), immunomodulation

## Abstract

Metastasis is the leading cause of cancer death worldwide. Circulating tumor cells (CTCs) are a critical step in the metastatic cascade and a good tool to study this process. We isolated CTCs from a syngeneic mouse model of hepatocellular carcinoma (HCC) and a human xenograft mouse model of castration-resistant prostate cancer (CRPC). From these models, novel primary tumor and CTC cell lines were established. CTCs exhibited greater migration than primary tumor-derived cells, as well as epithelial-to-mesenchymal transition (EMT), as observed from decreased E-cadherin and increased SLUG and fibronectin expression. Additionally, when fibronectin was knocked down in CTCs, integrin B1 and SLUG were decreased, indicating regulation of these molecules by fibronectin. Investigation of cell surface molecules and secreted cytokines conferring immunomodulatory advantage to CTCs revealed decreased major histocompatibility complex class I (MHCI) expression and decreased endostatin, C-X-C motif chemokine 5 (CXCL5), and proliferin secretion by CTCs. Taken together, these findings indicate that CTCs exhibit distinct characteristics from primary tumor-derived cells. Furthermore, CTCs demonstrate enhanced migration in part through fibronectin regulation of integrin B1 and SLUG. Further study of CTC biology will likely uncover additional important mechanisms of cancer metastasis.

## 1. Introduction

Metastasis is associated with advanced stages of cancer. Resulting in 90% of cancer deaths worldwide [1], metastasis occurs in a series of steps. These steps include the dissociation of cells from the primary tumor, migration through surrounding tissue, intravasation, circulation through blood, followed by extravasation and re-colonization of distant sites throughout the body. At advanced stages of most cancers, there are limited treatment options [1,2,3]. As such, efforts are increasingly being focused on identification of novel metastasis-related molecular targets.

One way to potentially avoid the need for invasive tissue biopsies when studying cancer metastasis is through the use of circulating tumor cells (CTCs). CTCs are cells which have dissociated from the primary tumor and are found traveling in the blood [4,5,6,7,8]. Some CTCs will eventually form metastatic, secondary lesions. Because CTCs can be obtained from liquid biopsies (from blood), they enable the molecular profiling of potentially unresectable tumors in patients [9] and identification of molecular changes important for progression to advanced cancers [10]. However, there is a challenge with low CTC numbers frequently found in the blood [11,12,13,14,15]. To address this potential obstacle to studying CTC biology, in this study, we established novel CTC cell lines and primary tumor-derived cells for molecular biological studies.

The two different cancer models used in this study were a syngeneic mouse model of hepatocellular carcinoma (HCC) and a xenograft mouse model of castration-resistant prostate cancer (CRPC). HCC is the most common form of liver cancer and is frequently diagnosed at very late stages. Consequently, it is one of the leading causes of cancer deaths worldwide [16,17,18,19]. Moreover, sorafenib, which is the main FDA approved drug to treat advanced HCC, extends life by only six months [20]. As such, better treatment options are needed. Similarly, CRPC is a form of prostate cancer (PCa) that is resistant to both medical and surgical castration [21,22]. However, androgen deprivation therapy (ADT) is the main standard of treatment for localized PCa [23,24]. This makes CRPC particularly challenging to treat. Over one third of CRPC patients will develop bone metastasis for which there is no cure [21,25]. Therefore, finding alternative treatments is critical for this cancer as well.

To this end, we propagated cell lines originating from primary tumors and CTCs. Our aim was to discover differences between these two cell types representing earlier and more advanced stages of cancers. Both HCC and CRPC CTCs demonstrate increased migration and evidence of epithelial-to-mesenchymal transition (EMT). Moreover, we discovered that in CTCs, fibronectin regulates integrin B1 and SLUG, which are known regulators of cell migration. Finally, we identified differences in CTC cell surface marker and cytokine secretion profiles that could have immunomodulatory implications. HCC CTCs had significantly reduced major histocompatibility complex class I (MHCI) expression, as well as significantly decreased secretion of endostatin, CXCL5, and proliferin as compared to primary tumor-derived cells. These findings may have implications for the function of metastatic cells and how they evade the immune system.

## 2. Materials and Methods

### 2.1. Cell Lines and Cell Culture

The BNL 1ME A 7R.1 cell line (purchased from ATCC), as well as the newly established primary tumor cell lines (TBOH1 and TBOH9) and circulating tumor cell lines (CBOH4 and CBOH9), were maintained in Dulbecco’s Modified Eagle Media (DMEM) media supplemented with 10% fetal bovine serum, 1% penicillin/streptomycin, and L-glutamine. Trypsinization of cells occurred using 0.25% trypsin when 75–80% confluent.

The 22Rv1 cell line (purchased from ATCC), as well as the newly established primary tumor cell line T22OH and circulating tumor cell line C22OH, were maintained in Roswell Park Memorial Institute (RPMI) media supplemented with 10% fetal bovine serum and 1% penicillin/streptomycin. Trypsinization of cells occurred using 0.05% trypsin when 75–80% confluent. All cells lines were cultured in a 5% CO_2_, 37 °C atmosphere.

### 2.2. Mouse Tumor Studies

**Figure 1:**
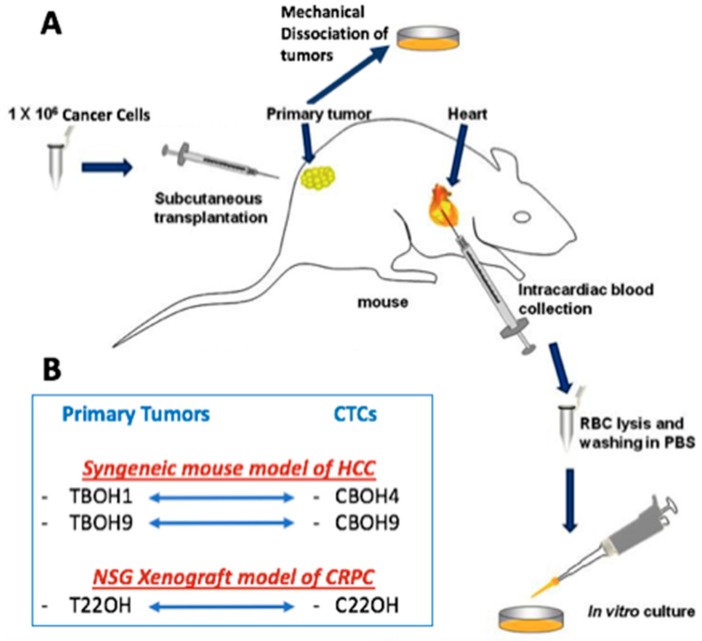
Establishment of novel primary tumor-derived cell lines and circulating tumor cell lines. (**A**) Schematic diagram summarizing how novel cell lines were established. This figure includes modifications to our previously described work [27]. For the syngeneic hepatocellular carcinoma (HCC) mouse model, BALB/c mice were subcutaneously implanted with the murine hepatocellular carcinoma cell line BNL 1ME A.7R.1. For the xenograft castration-resistant prostate cancer (CRPC) mouse model, NSG mice were subcutaneously implanted with the 22Rv1 human CRPC cell line. Mice were allowed to develop tumors over a period of 3–4 weeks, humanely sacrificed, primary tumors resected and mechanically dissociated, and then put in culture to establish the TBOH series of primary tumor-derived cell lines. The CBOH series of circulating tumor cells (CTCs) was established from cancer cells isolated from the bloodstream of the same mice implanted with either BNL 1ME A.7R.1 or 22Rv1 cells. (**B**) The newly established cell lines: the TBOH1 and CBOH4 pair and TBOH9 and CBOH9 pair were established from the HCC model. The T22OH and C22OH pair were established from the CRPC model.

Figure 1 shows the experimental mouse models used for this study and the subsequent establishment of novel cell lines. For the syngeneic mouse model of HCC, 7-week old, male, BALB/c mice were obtained from Taconic Biosciences Inc. Mice were implanted with 2.5 × 10^6^ BNL 1ME A 7R.1 murine HCC cells. For the NOD scid gamma (NSG) human xenograft mouse model, 7-week old, male NSG mice were obtained from Jackson Laboratory. Mice were implanted with 2.5 × 10^6^ 22Rv1 human CRPC cells. Tumors were allowed to grow until reaching a max tumor volume of 2000 cm^3^, at which point mice were euthanized, tumors removed, and samples of blood processed for CTCs as previously described by our lab [26]. Briefly, up to 1 mL of blood was obtained from intracardiac blood withdrawal from mice. The blood was spun down and the plasma removed. The rest of the blood sample, most importantly the buffy coat layer, which is where we expect our CTCs to be, was treated with red blood cell lysis buffer. After a series of spins and washes, samples were placed in media. Experiments on CTC cell lines were carried out for as long as forty-five passages (well over 6 months). Features remained consistent among different passages. While efficiency of cell line establishment was moderate to low, once established, the CTC cell lines exhibited high cell viability. In terms of our primary tumor cell lines, tumors were mechanically dissociated in media and given the chance to adhere to the plate to give rise to primary cell culture cell lines. H&E staining was performed to confirm metastasis to lungs using the core facilities at Albert Einstein School of Medicine. All mouse experiments were performed in compliance with Institutional Animal Care and Use Committee (IACUC)–approved protocols at Weill Cornell Medicine.

### 2.3. Immunofluorescence Staining

Cells were incubated with coverslips and grown for 48 hr. The coverslips were collected, fixed with 4% paraformaldehyde for 15 min at room temperature, permeabilized with 0.5% Triton X-100 in 1X PBS/1% FBS for 10 min at room temperature. Cells were stained with CREB3L3 antibody (sc-377331; 1:500; Santa Cruz Biotechnology, TX, USA), or endostatin antibody (PA1-601; 1:200; Thermo Fisher Scientific, Rockford, IL, USA) for 2 h, followed by incubation with Alexa Fluor 635 anti-rabbit secondary antibody (A31577; Thermo Fisher Scientific, Rockford, IL, USA) for 1 h. DAPI was used to counterstain nuclei, and slides were imaged using a Nikon A1 confocal microscope at a 60× magnification.

### 2.4. Migration Assays

For wound healing migration assays, 5 × 10^4^ murine BNL 1ME A.7R.1 cells were grown in a 6-well tissue culture plate and permitted to reach 90–100% confluency. Using a plastic tip (1 mm thick), wounds were administered to monolayers of cells in each well. Wounded monolayers were washed with 1X PBS and incubated with media. Cells were observed, and images were taken using the Motic AE30 Inverted Microscope.

For transwell migration assays, 1 × 10^5^ 22Rv1 CRPC cells were seeded on the top of chambers containing 8 μm pores (Greiner Bio-one, Austria, cat #: 662 638) in serum-free media. The bottom chambers were filled with regular media to serve as a chemoattractant. After 48 hr, the top chambers were rinsed with 1X PBS, fixed with paraformaldehyde, treated with methanol, and stained with trypan blue staining. The chambers were placed on a slide and viewed using the Motic AE30 Inverted Microscope. All migration assays were carried out at least 3 times.

### 2.5. Protein Extraction and Western Blotting

Whole cell extracts were obtained by treating cells with radioimmunoprecipitation assay (RIPA) lysis buffer (Amresco, Ohio, USA, cat#: N653), supplemented with 10× protease inhibitors (Thermo Fisher Scientific, Rockford, IL, USA, cat#: 88665) and 100 mM PMSF (Amresco, OH, USA, cat#: M145). Protein concentration was calculated via the Bradford Assay using the Bio-Rad Protein Assay Dye Reagent Concentrate. For Western blot analysis, 30 μg of protein was run on precast SDS-PAGE gels and subsequently transferred onto nitrocellulose membranes. Membranes were blocked in 5% BSA in TBS-T for 1 h at room temperature, incubated with primary antibodies overnight at 4 °C, washed with 1× TBS-T, incubated with secondary antibodies for 1 h, washed, and imaged using the LI-COR Odyssey CLx imager with infrared fluorescence. The primary antibodies used were directed against fibronectin (ab2413; 1:1000; Abcam, Cambridge, UK), E-cadherin (3195S; 1:200; Cell Signaling, MA, USA), CREB3L3 (sc-377331; 1:500; Santa Cruz Biotechnology, TX, USA), AR-V7 (ab198394; 1:1000; Abcam, Cambridge, UK), PSA (sc-7316; 1:200; Santa Cruz Biotechnology, TX, USA), integrin B1 (4706S; 1:500; Cell Signaling, MA, USA), GAPDH (5174S; 1:1000; Cell Signaling, MA, USA), alpha-tubulin (sc-32293; 1:500; Santa Cruz Biotechnology, TX, USA), and beta-actin (A5441; 1:5000; Sigma, St.Louis, MO, USA). The secondary antibodies used were anti-mouse (925-32210; 1:15,000; LI-COR, Lincoln, NE, USA) and anti-rabbit (925-32211; 1:15,000; LI-COR, Lincoln, NE, USA). Analysis and quantification of western blots were performed using ImageJ software.

### 2.6. RNA Extraction and qPCR Analysis

The RNeasy Mini Kit (QIAGEN, Hilden Germany, cat#: 74104) was used to isolate total RNA from each of the cell lines used in this study, according to the protocol specified by the manufacturer. RNA concentration was measured using the spectrophotometer NanoDropTM 2000 (Thermo Fisher Scientific, Inc.). cDNA was obtained using 1 μg of RNA and the QuantiTect Reverse Transcription kit (QIAGEN, Hilden, Germany, cat#: 205311).

Expression of SLUG was measured by quantitative real time qPCR using SYBR-Green Master mix (Life Technologies, CA, USA, cat#: 4309155). Primers for SLUG and GAPDH were created with the OligoPerfect Designer program (ThermoFisher Scientific Inc; Wilmington, DE, USA). The following oligonucleotide sequences were used for primers: murine SLUG-F, 5′-CCTTTCTCTTGCCCTCACTG-3′, and murine SLUG-R, 5′-ACAGCAGCCAGACTCCTCAT-3′; murine GAPDH-F, 5′-TGATGGGTGTGAACCACGAG-3′, and GAPDH-R, 5′-AGTGATGGCATGGACTGTGG-3′; human SLUG-F, 5′-CTTTTTCTTGCCCTCACTGC-3′, and human SLUG-R, 5′-GCTTCGGAGTGAAGAAATGC-3′; human GAPDH-F, 5′-GAGTCAACGGATTTGGTCGT-3′, and human GAPDH-R, 5′-TTGATTTTGGAGGGATCTCG-3′. For each sample, SLUG was normalized with GAPDH expression. The comparative cycle threshold (Ct) method was used to quantify relative target gene expression. The instrument used was the Quantifect Studio System (Applied Biosystems).

### 2.7. Transfection of siRNAs

Cells were grown in 6-well plates. When a confluency of 60–70% was reached, the cells were transfected according to the manufacturer’s instructions. Briefly, the cells were transfected with 10 nM of either fibronectin siRNA (Santa Cruz Biotechnology, TX, USA, cat#: sc-29315) or a non-targeting scramble control (Sigma, St.Louis, MO, USA) using Lipofectamine RNAiMAX (Thermo Fisher Scientific Inc.; Wilmington, DE, USA) diluted in Opti-MEM (ThermoFisher Scientific Inc.; Wilmington, DE, USA). The cells were then incubated for 24 h at 37 °C after which cells were harvested.

### 2.8. Flow Cytometry

Cells were incubated with trypsin for 3 min in order to harvest them. The pellets were washed, spun at 1200 rpm for 5 min, and re-suspended in 1% BSA in 1X PBS incubation buffer. Cells were spun and treated with mouse Fc block (BD Biosciences, NJ, USA, cat#: 553142) for 20 min. Either FITC anti-mouse MHCI antibody (BD Biosciences, NJ, USA, cat#: 553565) or its corresponding FITC Mouse Isotype control (BD Biosciences, NJ, USA, cat#: 553456) was added to cells and allowed to incubate for 1 h. The samples were fixed with 2% paraformaldehyde for 30 min at 4 °C, washed, and re-suspended in 1X PBS. Analysis was done using the BD FACs instrumentation and software version 7.0 (BD Biosciences, NJ, USA).

### 2.9. Cytokine Array

TBOH and CBOH cell lines were screened simultaneously for 111 murine cytokines using the Mouse XL Cytokine Array (R&D Biosystems, MN, USA, cat#: ARY028). Duplicate experiments were performed according to the manufacturer’s instructions. Cytokine signals were quantified with densitometry using Image J software.

### 2.10. Statistical Analyses

Data from at least 3 different independent experiments were collected and presented as mean ± standard error of the mean (SEM). Statistical significance was evaluated using Student’s *t*-test. p values of >0.05 were deemed significant.

## 3. Results

### 3.1. CTCs Obtained from Blood Express Tissue-Specific Markers

**Figure 2:**
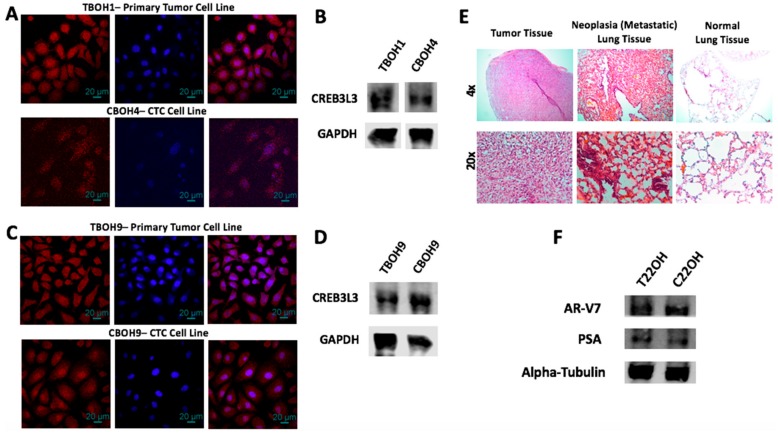
CTCs express tissue-specific markers and were obtained from mice that developed cancer metastasis. (**A**,**C**) Immunofluorescence staining for CREB3L3, a liver-specific marker; and (**B**,**D**) western blotting for CREB3L3. DAPI nuclear staining is shown in blue; CREB3L3 cytoplasmic staining is shown in red. (**E**) H&E staining of tumor and lung tissues from implantation of HCC cell line into BALB/c mice demonstrates evidence of cancer metastasis to lungs of cancer-bearing mice. (**F**) Western blotting was carried out to demonstrate prostate cancer origin for both CRPC primary tumor-derived and CTC lines. Experiments were carried out at least three times.

After the establishment of the novel cell lines (as shown in Figure 1), we confirmed tissue specificity of the established CTC lines. To confirm that the CTCs obtained from the bloodstream of the HCC syngeneic mouse models were of liver origin and not other cells potentially isolated from the blood, we performed immunofluorescence staining for CREB3L3, a validated liver specific marker [28,29]. As expected, the primary tumor cell lines TBOH1 and TBOH9 demonstrated strong CREB3L3 expression as shown by the red pigmentation in cells in Figure 2A,C. Similarly, the CTCs CBOH4 and CBOH9 also showed distinct CREB3L3 expression, confirming their liver origin, as well as their derivation from TBOH1 and TBOH9, respectively. No signal was observed in the negative controls in which cells were incubated with only the secondary antibody. Western blotting for CREB3L3 also revealed positive signals for both CBOH4 and CBOH9 as seen in Figure 2B,D.

In addition to showing that the CTCs are hepatic-specific, we also performed H&E histological staining on lung tissues obtained from the mice implanted with the HCC cell line. This was to confirm the occurrence of HCC metastasis to the lungs, as shown in Figure 2E by the darker red pigmentation in the lungs of the HCC-implanted mice in comparison to the lungs of the non-cancer bearing mice.

Similarly, to confirm that the cells obtained from the blood of the NSG mice implanted with CRPC cells are of prostate origin, we performed western blotting for prostate specific antigen (PSA) and the AR-V7 variant androgen receptor protein known to be expressed by 22Rv1. These are markers characteristic of prostate cancer cells [23,30,31]. Both the primary tumor-derived cell line and the CTC cell line derived from NSG mice implanted with the 22Rv1 CRPC cell line demonstrated distinct AR-V7 and PSA expression as shown in Figure 2F, confirming their origin from the prostate and the derivation of C22OH from T22OH. Furthermore, the occurrence of metastasis was confirmed by visually finding multiple macroscopic tumors in distant sites during necropsy of the mice.

Our results show that we were successfully able to isolate CTCs from the blood from the syngeneic HCC and NSG CRPC mouse models. Functional assays were subsequently carried out to determine differences between CTCs and their corresponding primary tumor-derived cells.

### 3.2. CTCs Have a Greater Migratory Capacity than Primary Tumor-Derived Cells

**Figure 3:**
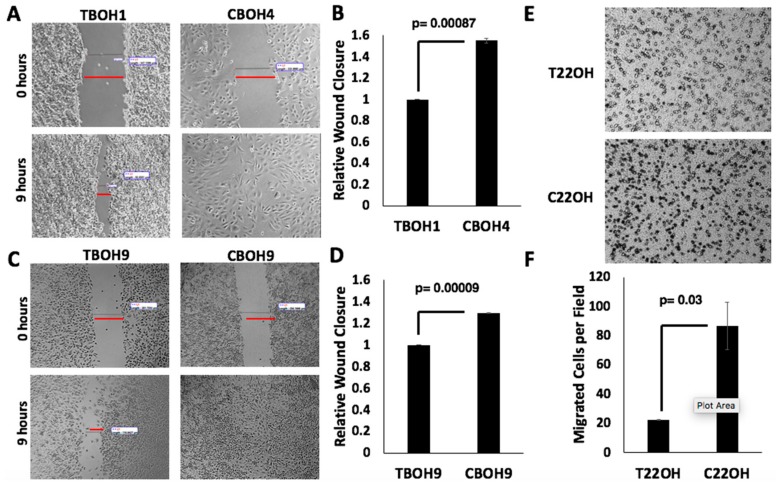
CTCs have greater migratory capacity than cancer cells from primary tumors. (**A**,**C**) Wound healing migration assays were performed on HCC cell lines. Cells were grown in 6-well plates. When confluent, wounds were made and measured at 0 and 9 h intervals. Wound closure by CTCs, but not by primary tumor-derived cell lines, was complete by 9 h. Images were taken at 10× magnification using Motic AE30 imaging software. (**B**,**D**) Migration (wound closure) was quantified; N = 3. (**E**) Transwell migration assays were performed on CRPC primary tumor-derived cell lines and CTC lines. Cells that migrated successfully through the pore membrane are represented by dark spots. Images shown were taken at 20× magnification. (**F**) Migration was quantified. Data provided on graphs are presented as mean ± standard error of the mean (SEM); N = 3.

Cancer cell migration is required for cancer metastasis [32,33]. To assess the migratory capability of both primary tumor-derived and CTC HCC cells, we performed wound healing migration assays. The rate at which wounds closed determined the migratory capability of cells. As shown in Figure 3A,C, we observed that CTCs (CBOH4 and CBOH9) were more migratory than their corresponding primary tumor-derived cell lines (TBOH1 and TBOH9, respectively). CBOH4 displayed a 55% increase in migration in comparison with its corresponding primary tumor-derived TBOH1 (Figure 3B), and CBOH9 demonstrated ~30% increased migration in comparison to TBOH9 (Figure 3D). The increased migratory capacity of CTCs is statistically significant.

To analyze the migratory capability of CRPC cells, we performed transwell migration assays. The number of cells passing through the chamber pores determined the migratory capability of cells. As shown in Figure 3E,F, the C220H CTC cell line demonstrated a four-fold increase in migration in comparison to the primary tumor-derived cell line T22OH.

### 3.3. CTCs Exhibit Epithelial to Mesenchymal Transition (EMT)

**Figure 4:**
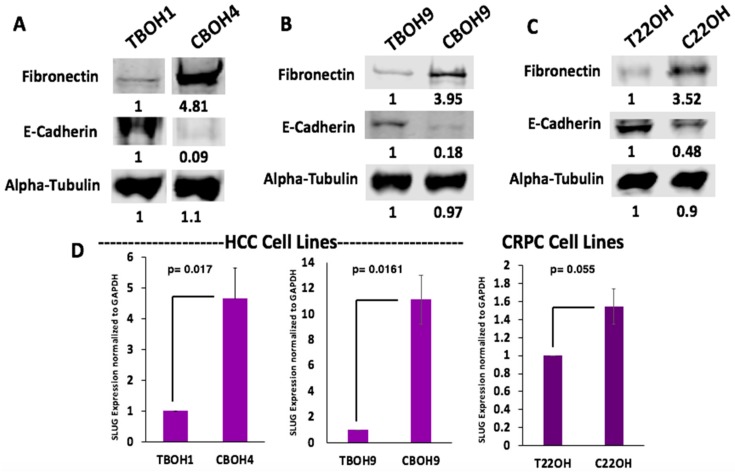
CTCs undergo epithelial-to-mesenchymal transition (EMT) as observed by increased fibronectin, decreased E-cadherin, and increased SLUG expression. (**A**,**B**) Fibronectin and E-cadherin protein expression by HCC primary tumor-derived cell lines and CTC lines. (**C**) Fibronectin and E-cadherin protein expression by CRPC primary tumor-derived cell line and CTC line. (**D**) SLUG expression was assessed using qPCR. Expression was normalized against GAPDH. Data provided on graphs are presented as mean ± standard error of the mean (SEM); N = 3.

Having observed greater migration from CTCs in comparison to primary tumor-derived cells, we wanted to determine whether CTCs were undergoing EMT. This is a phenomenon frequently observed in cancer cells migrating and metastasizing [34,35,36,37,38,39]. Using western blotting, we examined protein expression of fibronectin, a well-known marker of migratory and mesenchymal cells [40,41,42,43,44]. As observed in Figure 4A–C, CTCs had greater fibronectin protein expression in all three pairs of cell lines. More specifically, CBOH4 showed a 4.81-fold increase in expression of fibronectin when compared to TBOH1; CBOH9 exhibited a 3.95-fold increase in fibronectin expression when compared with TBOH9, and C22OH had a 3.52-fold increase in fibronectin expression in comparison with T22OH. Another marker examined to assess EMT was E-cadherin, a well-known cell adhesion protein characteristic of epithelial cells [45,46]. E-cadherin expression was decreased 11.1-fold in CBOH4 in comparison to TBOH1; decreased 5.5-fold in CBOH9 in comparison with TBOH9, and decreased 2.1-fold in C22OH in comparison with T22OH.

EMT is also made possible by several transcription factors that initiate and maintain it [47]. One such transcription factor observed to be overexpressed in all three CTC cell lines was SLUG. The mRNA expression of this EMT transcription factor was assessed using qPCR. As shown in Figure 4D, CBOH4 exhibited a 4.6-fold increase in SLUG mRNA expression in comparison with TBOH1. CBOH9 demonstrated an 11.1-fold increase in SLUG expression in comparison to the corresponding primary tumor-derived cell line TBOH9. Finally, C22OH exhibited a 1.5-fold increase in SLUG mRNA expression in comparison to the T22OH cell line. These findings demonstrate that EMT is occurring in CTC lines and may be the reason they are more migratory than primary tumor-derived cell lines.

### 3.4. Fibronectin Expression Regulates Integrin B1 and SLUG Expression in CTCs

**Figure 5:**
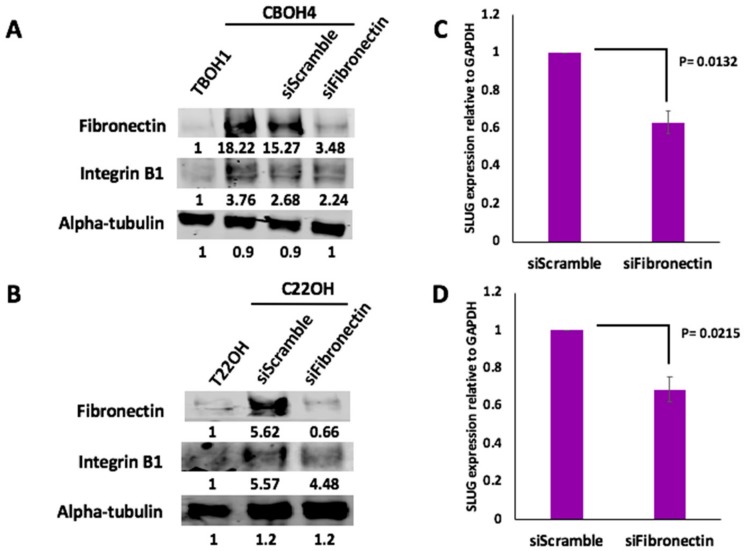
Fibronectin knockdown in CTCs caused decreased integrin B1 and SLUG expression. (**A**,**B**) After a 24 h transfection of CBOH4 and C22OH with fibronectin-specific siRNAs, western blotting was performed to determine the effects on integrin B1 expression. Western blotting revealed that fibronectin knockdown in CTCs was successful. The effect of fibronectin knockdown on integrin B1 expression was compared in control untransfected cells, CTCs transfected with scramble siRNAs, and CTCs transfected with fibronectin-specific siRNAs. Western blotting experiments were performed three separate times. (**C**,**D**) Effect of Fibronectin knockdown on SLUG expression in CBOH4 and C22OH CTCs was assessed by qPCR. Expression was normalized against GAPDH. Data provided on graphs are presented as mean ± standard error of the mean (SEM); N = 3.

Both HCC and CRPC CTCs expressed significantly more fibronectin than primary tumor-derived cells. We therefore investigated the molecular mechanisms of action of fibronectin in CTCs by knocking down fibronectin expression in CTCs. CBOH4 and C22OH were transfected for 24 h with either 25 pmol of fibronectin-specific siRNA or scramble siRNA. In comparison with primary tumor-derived cells, CTCs had higher expression of integrin B1 as shown in Figure 5A,B. Integrins are heterodimeric, transmembrane cell surface receptors that have been linked with metastasis and tumor migration [48,49,50]. The integrin B1 subunit specifically has been frequently upregulated in tumors [51,52]. Interestingly, knockdown of fibronectin in CTCs resulted in decreased integrin B1 expression (19.4% decrease in fibronectin expression in CBOH4 with knockdown of fibronectin; and 20.3% decrease in C22OH with knockdown of fibronectin).

Like integrin B1, SLUG is also a molecule that has been associated with greater migratory capacity by cancer cells [53,54,55]. To determine the effect of fibronectin knockdown on SLUG expression in CTCs, we performed qPCR analysis. As shown in Figure 5C, CBOH4 transfected with fibronectin-specific siRNA demonstrated a 40% decrease in SLUG expression in comparison with CBOH4 transfected with scramble siRNA. Similarly, as shown in Figure 5D, C22OH transfected with the fibronectin-specific siRNA demonstrated a 30% decrease in SLUG expression in comparison with C22OH transfected with scramble siRNA. Therefore, fibronectin has significant regulatory effects on integrin B1 and SLUG expression in CTCs.

### 3.5. HCC CTCs have Decreased MHCI Cell Surface Expression

**Figure 6:**
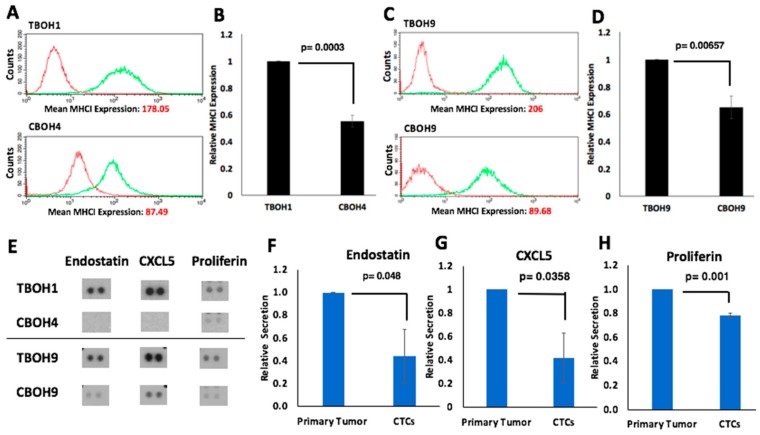
Immunomodulatory mechanisms of CTCs derived from a syngeneic mouse model of HCC. (**A–D**) Major histocompatibility complex class I (MHCI) expression was assessed using flow cytometry. CTCs have decreased MHCI cell surface protein expression in comparison to primary tumor-derived cell lines. Both isotype and MHCI antibodies were conjugated to fluorescein isothiocyanate (FITC). Isotype measuring background is shown in red. MHCI signal is shown in green; N = 5. (**E**) Analysis of 111 different cytokines secreted into cell media reveals consistent and significantly decreased secretion of endostatin, CXCL5, and proliferin in CTCs in comparison to primary tumor-derived cells; N = 2. (**F**–**H**) Endostatin, CXCL5, and proliferin signals from cytokine array were quantified. Data are presented as mean ± standard error of the mean (SEM).

To investigate potential immunomodulatory properties of CTCs, we used the cell lines derived from the syngeneic HCC mouse model. Using flow cytometry, we assayed for MHCI, a well-established cell surface molecule involved in self-recognition and identification of harmful entities for destruction by T-cells [56,57]. As shown in Figure 6A,B, we observed a 1.8-fold (45%) decrease in MHCI expression in the CBOH4 CTC line in comparison to the primary-tumor derived TBOH1 cell line. Similarly, as shown in Figure 6C,D, we observed a 1.5-fold (35%) decrease in MHCI expression in the CBOH9 CTC line in comparison to the TBOH9 primary tumor-derived cell line.

### 3.6. HCC CTCs have Significantly Decreased Secretion of Endostatin, CXCL5, and Proliferin

Cytokines are secreted molecules that can activate signaling pathways and have effects on the immune response [58,59,60]. To determine if there are differences in the cytokine secretion profile between CTCs and primary tumor-derived cells, a cytokine array was used. Of the 111 cytokines assayed, three were consistently and significantly lower in CTCs (see Figure 6E). Endostatin, an anti-angiogenic molecule and inhibitor of tumor growth [61,62], was decreased 2.27-fold in CTCs in comparison to primary tumor-derived cells (Figure 6F). CXCL5, a molecule that plays a role in attracting leukocytes such as neutrophils [63,64], was also downregulated 2.38-fold in CTCs (Figure 6G). Finally, proliferin, a molecule with reported roles in cell growth regulation and differentiation [65,66,67], was decreased 1.28-fold in CTCs (Figure 6H). Statistical analyses found all three molecules to be significantly decreased with p values of <0.05.

### 3.7. CTCs, in Comparison to Primary Tumor-Derived Cells, Have Decreased Endostatin Expression

**Figure 7:**
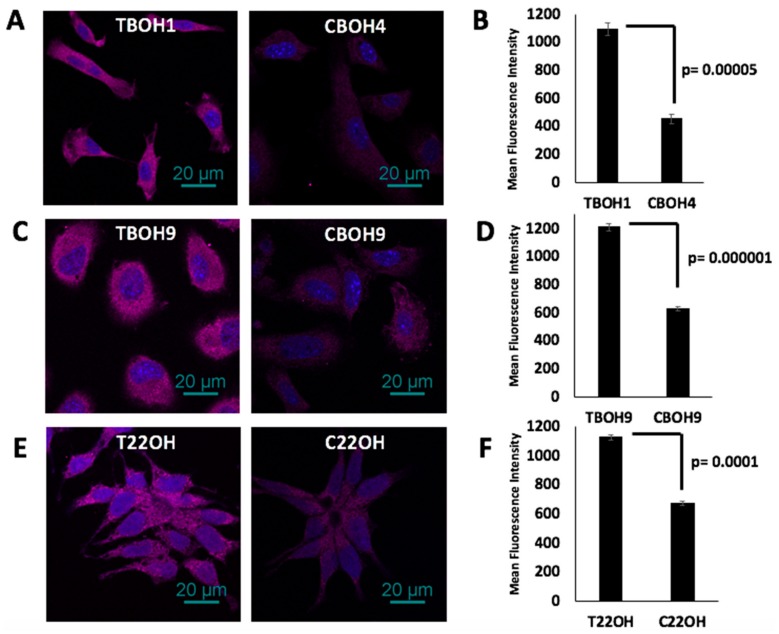
Decreased intracellular endostatin expression by CTCs. (**A**,**C**,**E**) Endostatin expression in TBOH1 and CBOH4, TBOH9 and CBOH9, and T22OH and C22OH was determined using immunofluorescence. (**B**,**D**,**F**) Mean fluorescence intensity was quantified using the NIS Elements software. Data are presented as mean ± standard error of the mean (SEM).

As a follow-up to the observation that CTCs secreted significantly reduced endostatin, we investigated intracellular endostatin expression in both of our HCC and CRPC CTC models using quantitative immunofluorescence. As shown in Figure 7A,B, CBOH4 had a 59% decrease in endostatin expression in comparison to TBOH1, and as shown in Figure 7C,D, CBOH9 had a 48% decrease in endostatin expression compared to TBOH9. Further, as shown in Figure 7E,F, C22OH had a 40% decrease in the expression of endostatin in comparison to T22OH. Thus, we can conclude that CTCs from both HCC and CRPC models examined expressed significantly less intracellular endostatin than their corresponding primary tumor-derived cells.

## 4. Discussion

Metastasis is the most common cause of death among cancer patients. Molecular mechanisms of cancer metastasis are not yet clear [1,2,3,33,68]. CTCs are an important step in the metastatic process. Consequently, in this study, we have focused on elucidating the molecular mechanisms of CTCs.

However, CTCs are not always abundant and are usually challenging to obtain in the human clinical setting [12,13,14,15]. To address these limitations, we have established an effective method for isolating CTCs, establishing long-term cultures, and propagating them into cell lines that are useful for studying CTC biology. The challenge involved in this task is underscored by the fact that only a few other groups have had success in doing this [69,70,71,72]. So far, it has been easier to establish short term CTC cultures, which when coupled with single-cell sequencing or short-term biochemical assays, have resulted in very useful information [73,74,75,76,77,78,79,80,81,82]. Further, these techniques can be physically intensive and expensive. Here, we have described an approach for propagating CTC cell lines and using them for functional characterization, identification of novel molecular pathways important to metastasis, as well as to gain insight into molecular receptors and secretions that might have immunomodulatory functions in CTCs.

In this study, we have focused on the use of HCC and CRPC models because they both have significant negative clinical outcomes. At present, there is no effective treatment for advanced HCC [16,17,18,20]. Similarly, metastatic CRPC currently has no cure [21,22,23,24,83].

In the present study, we have successfully propagated three pairs of CTC lines and their corresponding primary tumor-derived cell lines: two pairs of HCC origin and one pair of CRPC origin. Interestingly, all CTCs demonstrated significantly greater migratory capacity than their primary tumor-derived counterparts. Furthermore, we investigated the role of EMT in CTCs. EMT is a phenomenon that is frequently observed in cancer cells during cancer progression [34,35,36]. Accordingly, we observed evidence of EMT in CTCs as demonstrated by downregulation of E-cadherin and upregulation of fibronectin and SLUG expression. Therefore, our findings indicate that enhanced migratory capacity and EMT are characteristic of CTCs.

Because we observed fibronectin to be overexpressed by CTCs, we were also interested in identifying molecules regulated by fibronectin. Integrin receptors have long been associated with tumor migration [49]. While fibronectin binding and activation of these receptors is established [84,85], we report for the first time our observation that fibronectin has a regulatory role in the expression of the integrin B1 subunit. Fibronectin knockdown in CTCs resulted in decreased expression of integrin B1. Interestingly, another molecule significantly reduced by fibronectin knockdown in CTCs is SLUG. SLUG is a known regulator of EMT, and we observed SLUG overexpression in CTCs in comparison to primary tumor-derived cells. Upon fibronectin knockdown, SLUG expression was significantly decreased in CTCs. Thus, fibronectin expression regulates integrin B1 and SLUG expression in CTCs.

In this study, we also investigated major histocompatibility complex class I (MHCI) expression and secretion of cytokines by CTCs. The major histocompatibility complex class I (MHCI) is an important cell surface molecule that enables T cells to distinguish between “self” vs. “non-self”. Upon recognition of harmful entities presented by MHCI, T cells will attack these MHCI-presenting cells [56,57]. We observed significant reduction of MHCI expression in CTCs arising from the syngeneic HCC mouse model. This has interesting implications on how CTCs may circumvent immunosurveillance. Additionally, we sought to identify molecules differentially secreted by CTCs in comparison to primary tumors. To this end, we assayed over 111 different cytokines using media secreted by cells. We found three molecules to be significantly decreased by CTCs: endostatin, CXCL5, and proliferin. We report for the first time that endostatin, CXCL5, and proliferin are significantly secreted less by CTCs than primary tumor-derived cells. Further studies are imperative to clarify the implications of this differential secretion profile of CTCs to cancer metastasis.

Endostatin is known for its anti-angiogenic properties [61,62,86]. The fact that CTCs express less endostatin is intriguing and suggests that CTCs have acquired enhanced angiogenicity, which bodes well for the colonization of secondary sites. Moreover, it is conceivable that decreased endostatin secretion by CTCs may enhance their metastatic capability in other important ways [87,88,89]. CXCL5 is a chemokine that recruits and activates neutrophils. While some studies associate higher CXCL5 expression with a worse cancer prognosis [63,64,90,91], several reports indicate that a lower CXCL5 expression can also promote metastatic spread [91,92,93,94]. Proliferin is a placental growth hormone that has been observed to be pro-angiogenic [65,66,67]. The implications of our current finding that CTCs secrete reduced amounts of proliferin than primary tumor-derived cells are currently unclear and deserve further study. It is noteworthy, though, that previous studies have found that as tumors grow in volume, intra-tumoral cells become hypoxic due to lack of oxygen within the tumor mass. This, in turn, stimulates the production of pro-angiogenic factors, enabling new blood vessel formation inside the tumor to deliver oxygen [95]. This process may not be necessary for CTCs, and this may explain the reduced secretion of proliferin by CTCs observed.

Interestingly, endostatin expression was found to be downregulated in all our CTCs regardless of tissue of origin. This was even more compelling since fibronectin, which was upregulated in our CTCs, has been previously reported to bind to the same integrin α5β1 receptor as does endostatin [85,96,97,98,99,100]. It is plausible that there could be a potential autocrine, competitive binding occurring between fibronectin and endostatin to the integrin α5β1 receptor with resulting implications in a cancer cell’s ability to migrate. Further studies are necessary to confirm or refute this.

It is important to also note that while our work highlights a novel methodology and the creation of new cell lines to uncover possible mechanisms of cancer cell action, our cell lines were obtained from murine models. Others have previously isolated CTCs from human patients using microfluidic devices to assess molecular phenotype and drug sensitivity [9,69,70,71,72]. We propose our methodology to contribute to the field by adding yet another way in which we can harness the information gained from studying CTCs. In doing so, we may refute or support novel ideas such as CTCs being hybrids between leukocytes and cancer cells [101,102]. Whether this explains the reason our CTCs demonstrate enhanced migration and higher levels of integrin B1, which was previously reported in leukocytes [103,104], remains to be proven.

In summary, we have described the establishment of novel primary tumor-derived cell lines and CTC lines from the same mouse models and have used them to elucidate novel molecular mechanisms of CTCs. This work has demonstrated that EMT, enhanced migration, and decreased endostatin, CXCL5, and proliferin secretion are consistently seen in CTCs. Additionally, we report a novel role for fibronectin in regulating integrin B1 subunit receptor expression in CTCs, and while fibronectin’s ability to regulate SLUG activity in renal cell carcinoma cells to promote lung metastasis has been reported [105], we report for the first time an observation of this molecular phenomenon in CTCs of HCC and CRPC origin. Our findings suggest that fibronectin’s regulation of SLUG in CTCs may contribute to their role in cancer metastasis. Taken together, our findings demonstrate that CTCs have unique and important molecular mechanisms with implications for cancer metastasis.

## 5. Conclusions

In conclusion, we have demonstrated the successful establishment of novel primary tumor-derived cell lines and CTC lines from a syngeneic HCC mouse model and a human xenograft CRPC mouse model. The CTCs in these models demonstrate enhanced migration and EMT when compared to primary tumor-derived cells. Here, we report our novel finding of fibronectin’s regulation of integrin B1 and SLUG expression in CTCs. This may be a mechanism by which CTCs ensure greater migration and metastasis. Further, we report our observation of decreased MHCI expression and decreased secretion of endostatin, CXCL5, and proliferin by CTCs in comparison to primary tumor-derived cells. These molecular mechanisms of CTCs likely have important implications for cancer metastasis.
